# Episodic Future Thinking about the Ideal Self Induces Lower Discounting, Leading to a Decreased Tendency toward Cheating

**DOI:** 10.3389/fpsyg.2017.00287

**Published:** 2017-03-02

**Authors:** Wen-Hsiung Wu, Wen Cheng, Wen-Bin Chiou

**Affiliations:** ^1^Department of Healthcare Administration and Medical Informatics, Kaohsiung Medical UniversityKaohsiung, Taiwan; ^2^Center for Teacher Education, National Sun Yat-sen UniversityKaohsiung, Taiwan; ^3^Institute of Education, National Sun Yat-sen UniversityKaohsiung, Taiwan

**Keywords:** cheating, delay discounting, delinquency, episodic future thinking, ideal self, morality

## Abstract

Delay discounting refers to a pervasive tendency toward preferring smaller immediate gains over larger future gains. Recent empirical research has shown that episodic future thinking (EFT; i.e., projecting oneself into the future to pre-experience forthcoming events) can reduce the tendency toward discounting. A common tenet of psychological theories of crime is that delinquency results from focusing on short-term gains while failing to consider adequately the longer-term consequences of delinquent behavior. We investigated whether an EFT intervention involving the ideal self could induce lower discounting rates and, as a consequence, reduced delinquency. The results showed that, compared with control participants, participants engaging in EFT, that is, envisaging life events that would be experienced by their ideal selves, exhibited a lower discounting rate in a monetary choice task (Experiments 1 and 2), as well as a decreased tendency to make delinquent choices in imaginary scenarios (Experiment 1) and cheat in a matrix task (Experiment 2). The discounting tendency mediated the relationship between engaging in EFT pertaining to the ideal self and the tendency toward morally questionable behavior (Experiments 1 and 2). The findings of the two experiments indicate that engagement in EFT with a focus on the ideal self is sufficient to induce lower discounting rates, by promoting consideration of distant costs and thus increasing resistance to delinquent involvement and cheating (given the temptation of the immediate benefits that may accrue from such behavior). The current research constitutes an innovative approach to delinquency prevention and the promotion of morality.

## Introduction

Delay discounting, also known as time discounting, refers to a preference for smaller immediate rewards over larger delayed ones (Kirby and Maraković, 1995; Frederick et al., 2002). The discounting tendency has been considered as a key tenet of several established theories and explanations of crime; the tendency to live in the here and now plays a major role in delinquency by interfering with the capability to focus on the long-term consequences of one’s actions (Gottfredson and Hirschi, 1990; Moffitt, 1993; Pratt and Cullen, 2000; Nagin and Pogarsky, 2003; van Gelder et al., 2013). Thus, the tendency to delay gratification (i.e., lower discounting rate) could be a crucial determinant of delinquency. Recent advances in techniques to decrease the discounting tendency indicate that engagement in *episodic future thinking* (EFT; projecting oneself into the future to pre-experience forthcoming events; Atance and O’Neill, 2001; Schacter et al., 2007) can result in lower discounting rates (Peters and Büchel, 2010; Benoit et al., 2011; Cheng et al., 2012; Gellert et al., 2012; Daniel et al., 2013a,b; Lin and Epstein, 2014). Self-concept theories suggest that activation of the ideal self (i.e., an ideal version of one’s future self; Markus and Nurius, 1986) may promote goal attainment (Carver and Scheier, 1999; Gollwitzer, 1999; Freitas and Higgins, 2002). The present study provides experimental evidence that an EFT intervention involving the ideal self is associated with lower discounting, a decreased tendency toward virtual delinquent choices, and a reduced likelihood of cheating.

### Episodic Future Thinking and Delay Discounting

Episodic future thinking principally involves vivid mental simulation of future experiences, whereas *semantic future thinking* (SFT) may be broadly defined as general knowledge regarding the future (Atance and O’Neill, 2001; Schacter et al., 2007). As an example, the episodic/semantic distinction is captured by the difference between envisioning specific events occurring during a forthcoming vacation (episodic) versus having knowledge regarding a particularly scenic spot (semantic). Given that EFT projects the self into pre-experiencing future events, engagement in EFT may activate brain regions involved in prospective thinking (Schacter et al., 2007), thereby inducing a future orientation (Atance and O’Neill, 2001). Hence, the delay discounting-reducing effect of EFT results from the ability of prospective images to enhance either considerations of distant rewards (Benoit et al., 2011) or efforts to determine the value of delayed outcomes (Kurth-Nelson et al., 2012) in intertemporal choices. Prior research has demonstrated that EFT can reduce the tendency to discount the future (e.g., Peters and Büchel, 2010; Cheng et al., 2012; Daniel et al., 2013a,b). For example, Peters and Büchel (2010) showed that engagement in EFT induced a lower discounting rate in the context of intertemporal choice. Similarly, Cheng et al. (2012) demonstrated that participants engaging in prospective imagery showed an increased focus on future gains, in the context of financial decision-making, compared with control participants. Daniel et al. (2013a) provided experimental evidence that overweight and obese women engaging in EFT showed lower discounting rates. Daniel et al. (2013b) also showed that EFT was effective in reducing discounting tendencies in both obese and lean individuals. EFT interventions may decrease the discounting tendency by activating a future-oriented mindset (Schacter et al., 2007; Cheng et al., 2012; Chiou and Wu, 2017).

Self-theorists suggest that prospective thinking pertaining to *the ideal self* (i.e., an ideal version of one’s future self; Markus and Nurius, 1986) can facilitate self-regulatory efforts (Carver and Scheier, 1999). Individuals may be motivated to engage in goal-directed behaviors that aid in achieving the desired state when envisaging the ideal self (Higgins, 1996). In other words, the ideal self may motivate one to engage in self-regulatory behavior to achieve desirable aspects of the self (Freitas and Higgins, 2002). However, SFT involves only semantic knowledge about the future (Atance and O’Neill, 2001). Atance and O’Neill (2001) pointed out that the relationship between implementation intentions and goal attainment is mediated by EFT, but not by SFT. For instance, participants who were instructed merely to list positive aspects of their desired future failed to commit to goals (Oettingen, 1996, 2000; Oettingen et al., 2001). Compared with participants whose only goal was to take a vitamin C pill (SFT manipulation), those who were required to commit themselves to taking the pill at a particular time and place every day (EFT manipulation) missed fewer doses after both 10 days and 3 weeks (Sheeran and Orbell, 1999).

In principle, the ideal self involves images of a desired future (Boyatzis and Akrivou, 2006). This image is the ideal version of one’s future self (Markus and Nurius, 1986). Being primed with the ideal self may induce a future-oriented mindset, which facilitates considerations of future consequences and thereby reduces delay discounting (Chen and Vazsonyi, 2011; Cheng et al., 2012). Thus, thinking about one’s ideal self may activate a mindset that includes a future orientation, and which, thereby, reinforces tendencies toward delayed gratification. Two recent studies showed that interacting with a virtual lower-weight self (i.e., the ideal self of participants who were intending to lose weight) induced lower discounting rates (Kuo et al., 2016), while imagining future life events involving a non-smoking self (i.e., the ideal self of smokers intending to quit or reduce smoking) reduced the tendency toward discounting (Chiou and Wu, 2017). Moreover, EFT about positive events was associated with increased clarity about temporal considerations and more vivid pre-experiencing than was EFT about negative events (D’Argembeau and Van der Linden, 2004). A fundamental tendency shared by most individuals is the preferential processing of information that conveys a positive view of themselves (Taylor and Brown, 1988; Baumeister, 1998). Given that the ideal self represents a positive, desirable version of the future self, and given that the generation of possible future events is influenced by self-relevant goals (Johnson and Sherman, 1990), engagement in EFT about the future ideal self should be more likely to induce a future orientation than engagement in EFT about the future more generally. Individuals may generate images of positive or negative life events when engaging in EFT about the future in general. Taken together, the aforementioned theoretical considerations and findings suggest that an EFT intervention focusing on the ideal self would be more effective in reducing the discounting tendency than would SFT and EFT about the future more generally.

### Delay Discounting and Delinquency

From the perspective of the association between delay discounting and crime, delinquency entails a typical intertemporal choice dilemma involving the immediate gains of delinquent behavior versus distant costs (e.g., criminal record, loss of studentship or vocation, or social ostracism). Delinquent acts may tempt people into focusing on short-term gains, such as money, sexual gratification, or excitement, but incur greater long-term costs (Hirschi, 2004). The extent to which individuals focus on the remote costs of delinquency is crucial in determining whether they will choose to commit a delinquent act or abstain from crime (Wilson and Herrnstein, 1985). Hence, the tendency to discount the future can lead to delinquency by interfering with the ability to think through the future consequences of delinquent behavior (Moffitt, 1993).

In a study by Gottfredson and Hirschi (1990), people who showed greater discounting had a reduced ability to defer gratification, leading them to engage in criminal activity. In a similar vein, Nagin and Pogarsky (2003) proposed that individuals with high discounting rates are less deterred by the delayed cost of their behaviors. Moreover, neuroimaging evidence has shown that both delay discounting (Harris et al., 2013; Hare et al., 2014) and morally questionable behavior (Greene and Paxton, 2009; Greene and Haidt, 2012) are associated with activity in prefrontal cortical regions implicated in self-control. Given that delinquency results from the inability to make deliberate, trade-off-based decisions that weigh immediate gains against the distant costs of delinquent behavior, any reduction in the tendency toward discounting induced by EFT (i.e., imagining future life events experienced by the ideal self) may in turn lower the tendency toward delinquent commitment.

### Overview of the Present Research

Building on prior work showing that EFT can induce lower discounting rates (Peters and Büchel, 2010; Cheng et al., 2012; Daniel et al., 2013a,b; Chiou and Wu, 2017), and on the notion that a lower tendency toward discounting is associated with decreased involvement in delinquency (Gottfredson and Hirschi, 1990; Moffitt, 1993; Pratt and Cullen, 2000; Nagin and Pogarsky, 2003), we conducted two experiments to test whether engaging in prospective imagery, to pre-experience the life events that would be experienced by the ideal self, leads to lower discounting rates, thereby reducing the likelihood of making delinquent choices (Experiment 1) or cheating (Experiment 2).

## Experiment 1: EFT Pertaining to the Ideal Self, Discounting, and Delinquent Choices

### Method

A total of 90 undergraduate students (42 females and 48 males; mean age = 20.9 ± 1.4 years) enrolled at a public university in southern Taiwan participated in this experiment for extra course credit. The sample size was determined by calculating the number of participants required to satisfy the omnibus *F*-test (number of groups = 3) under the following conditions: α = 0.05; ω^2^ = 0.10 (a small association; Cohen, 1988); and power (1 - β) = 0.80 (Kirk, 2012; p. 925).

Upon arrival at the laboratory, the participants were informed that they would be assisting with the piloting of several unrelated tasks that would be used in future studies. After providing written informed consent, every three participants were randomly assigned to one of the three study conditions (EFT, SFT, and control) via a permuted-block randomization schedule. Under the EFT condition, participants were instructed to write down three aspects of their ideal selves (e.g., physical, social, moral, and psychological) and then listed three life events that would occur “if the desirable aspects of the self are realized in 1 year’s time.” The 1-year period was identical to that of a subsequent delay-discounting task. The participants were then asked to close their eyes and to try to imagine the events they had listed as specifically and vividly as possible (i.e., to imagine the setting and sequence of the events, the persons and objects that would be present, etc.; D’Argembeau and Van der Linden, 2004). They had 1 min to pre-experience each life event mentally. In contrast with the EFT condition, participants under the SFT condition were instructed to write about only three events related to aspects of their ideal selves (e.g., physical, social, moral, and psychological) that would occur within 1 year. Therefore, participants in the SFT group generated representations of only their ideal selves. They did not engage in prospective imagery to mentally pre-experience the life events of the ideal self. Each participant in the SFT condition was given the same amount of time as the matched participant in the EFT condition. In accordance with the yoking procedure, the mean time spent performing the manipulation task was approximately equal between the EFT and SFT conditions. Participants under the control condition completed an unrelated questionnaire (e.g., Freitas et al., 2004; Chiou et al., 2013; Chiou and Wu, 2017; the Big Five Inventory in the current study, John and Srivastava, 1999) requiring approximately 6–10 min of their time.

The thinking manipulation task was followed by a delay discounting task, in which participants indicated their preferences in relation to winning the lottery; they made nine binary choices between either receiving $120 immediately or varying amounts of money ($113, $120, $137, $154, $171, $189, $206, $223, and $240) (Hardisty and Weber, 2009) in 1 year. To increase involvement in the discounting task, participants were told that one of them would receive a free gift certificate to a major online retailer (Joshi and Fast, 2013). They were further informed that “what you would receive is determined by random selection of one of the nine choices you make.” We calculated the discount rate, *k*, by employing the hyperbolic-discounting formula (see Hardisty and Weber, 2009). Larger values of *k* imply greater temporal discounting.

The final task was a questionnaire comprising six delinquent choice scenarios, which were presented according to how often they might be encountered in real life (van Gelder et al., 2013; Donner et al., 2014). For example, a scenario pertaining to the purchase of stolen goods online read as follows:

*Imagine the following*: You want the latest mobile phone but are short on cash. A fellow student forward you a link to an Internet vendor who is selling the latest models of mobile phones that ‘fell off a shipping truck.’ The mobile phones are very attractively priced. How likely is it that you would buy a potentially stolen mobile phone?

The other scenarios concerned cyberbullying (posting hurtful information online), fraud, illegal downloading, purchasing counterfeit goods, and theft. Participants recorded their responses on a 7-point scale (from *very unlikely* to *very likely*). The responses to the six scenarios were averaged to form a delinquent choice index (α = 0.86; *M* = 3.86, *SD* = 1.27). Higher scores on this index reflect a tendency to make more delinquent choices. At the end of the experiment, a probe for suspicion was administered to each participant. None of the participants accurately guessed as to how the three tasks were related.

### Results

As predicted, the discounting tendency was associated with the thinking manipulation [*F*_(2,87)_ = 5.807, *p* = 0.004, $\eta_{p}^{2}$ = 0.118; **Table 1**]. Follow-up contrasts showed that participants in the EFT group discounted less than did those in the SFT (*t* = -2.449, *p* = 0.016, Cohen’s *d* = 0.63) and control (*t* = -3.277, *p* = 0.002, *d* = 0.85) groups. The discounting rate did not differ between the SFT and control groups (*t* = -0.828, *p* = 0.41). In addition, the discounting rate did not differ between male (*M* = 0.56, *SD* = 0.24) and female (*M* = 0.52, *SD* = 0.25) participants [*F*_(1,84)_ = 0.645, *p* = 0.424]. The interaction of condition by participant sex was not significant [*F*_(2,84)_ = 0.329, *p* = 0.721].

**Table 1:** Descriptive statistics by condition in Experiment 1.

Measure	EFT		SFT		Control	
			
	*M*	*SD*	*M*	*SD*	*M*	*SD*
Discounting rate	0.43	0.23	0.58	0.22	0.62	0.22
Delinquent-choice index	3.36	1.21	4.05	1.35	4.17	1.12


*Each condition involved 30 participants. Smaller values of the discounting rate indicate lower discounting. The delinquent-choice index ranged from 1 to 7. Higher scores indicate a higher likelihood of choosing delinquent options in imaginary scenarios (e.g., buying stolen goods online, cyberbullying, illegal downloading, purchasing counterfeits, and theft). Both the EFT and SFT conditions focused on the ideal self. EFT, episodic future thinking; SFT, semantic future thinking.*

As shown in **Table 1**, the delinquent-choice index differed among the three study conditions [*F*_(2,87)_ = 3.815, *p* = 0.026, $\eta_{p}^{2}$ = 0.081]. Participants under the EFT condition scored significantly lower on the delinquent-choice index than did those under the SFT (*t* = -2.187, *p* = 0.031, *d* = 0.56) and control (*t* = -2.555, *p* = 0.012, *d* = 0.66) conditions. In addition, the delinquent-choice index did not differ between male (*M* = 3.89, *SD* = 1.36) and female (*M* = 3.83, *SD* = 1.17) participants [*F*_(1,84)_ = 0.052, *p* = 0.819]. The experimental effect did not interact with participant sex [*F*_(2,84)_ = 0.698, *p* = 0.501].

**FIGURE 1:**
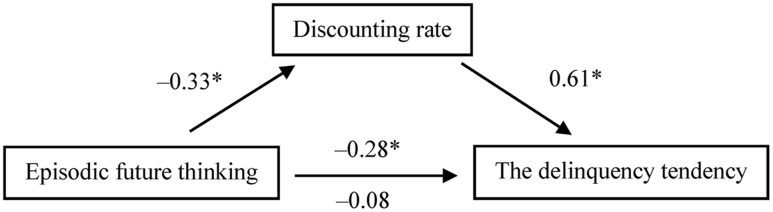
**Mediation of the effect of thinking manipulation (1 = episodic future thinking, 0 = semantic future thinking and control) on the tendency to make virtual delinquent choices in Experiment 1.** Values are standardized regression coefficients. Smaller values of the discounting rate indicate lower discounting. On the lower path, the values below and above the arrow are the results of analyses in which the mediator was and was not included in the model, respectively. Asterisks indicate significant path coefficients (^∗^*p* < 0.05).

Moreover, we examined whether delay discounting mediated the link between the thinking manipulation and the delinquency tendency. As the SFT and control groups did not differ in the dependent measures, these two groups were combined as the reference group for the dummy variable (1 = EFT, 0 = SFT, and control). A bootstrap analysis (Preacher and Hayes, 2004) showed that the indirect effect was significant [*B* = -0.54, *SE* = 0.18, 95% bias-corrected confidence interval (CI): -0.97 to -0.22; bootstrap resamples = 5000; **Figure 1**]. Engagement in EFT predicted the discounting rate (*B* = -0.17, *SE* = 0.05, *t* = -3.312, *p* = 0.001), the discounting rate predicted the delinquent-choice index (*B* = 3.27, *SE* = 0.47, *t* = 6.987, *p* < 0.001), and the connection between engagement in EFT and the delinquent-choice index (*B* = -0.75, *SE* = 0.27, *t* = -2.751, *p* = 0.007) was no longer significant (*B* = -0.21, *SE* = 0.23, *t* = -0.897, *p* = 0.372) when we controlled for the discounting rate. Thus, the mediation analysis results suggest that lower discounting, induced by engaging in EFT to envisage life events of the ideal self, leads to a reduced tendency toward virtual delinquent choices.

### Discussion Experiment 1

The results indicated that engaging in EFT pertaining to the ideal self led to lower discounting, which did not appear to be the case with SFT. Among our participants, envisaging future life events experienced by the ideal self influenced the tendency to make delinquent choices in imaginary scenarios. It should be noted that the inclusion of an SFT condition ruled out the possibility that differences in the dependent measures between the SFT and EFT conditions were due simply to thinking ahead. In other words, engagement in SFT about the ideal self was not sufficient to produce the reduction effects on discounting and delinquent choices. Furthermore, the control group was not subject to any thinking manipulation. Whether merely engaging in episodic thinking (ET) about one’s current experiences would also be sufficient to produce a similar reduction in delinquency remains unknown. Thus, a control ET condition and an ET condition pertaining to the present self were included in Experiment 2. Additionally, given that the tendency toward delinquent choices relied on a self-report measure and lacked a behavioral indicator, a behavioral measure was also employed in Experiment 2.

## Experiment 2: EFT Pertaining to the Ideal Self, Discounting, and Cheating

### Method

In total, 90 undergraduate students (45 females and 45 males; mean age = 20.2 ± 2.0 years) were recruited via posters from a public university in southern Taiwan. The sample size was determined according to the number of participants required to satisfy the omnibus *F*-test (number of groups = 3) under the following conditions: α = 0.05, ω^2^ = 0.10, and power = 0.80. Thus, the required sample size was 90 (Kirk, 2012; p. 925).

Every three same-sex participants were randomly assigned to one of the three thinking conditions (EFT pertaining to the ideal self, control ET, and ET pertaining to the present self). The sex ratios were identical between the groups. Under the EFT ideal-self condition, participants engaged with the same thinking exercise (i.e., identical procedures and materials) that was employed in Experiment 1. Under the control ET condition, participants were instructed to read about three life events taken from the personal blog of a travel writer, and then engaged in ET to pre-experience non-personal autobiographical details mentally (e.g., Daniel et al., 2013a). Under the ET present-self condition, participants were instructed to list three routine life events that occurred in their present-day life, and then engaged in ET to re-experience these events. The mean time spent performing the ET task was approximately equal among the three study conditions. At the end of the thinking exercise, participants rated their simulations in terms of contextual detail and vividness of imagery using Likert-type scales (1 = *not at all*, 7 = *very much*).

After the thinking exercise, each participant completed an ostensibly unrelated survey measuring discounting tendency. The discounting measure was identical to that of Experiment 1. Under the auspices of pilot testing a future experiment, the participants were then given a worksheet of 20 number matrices; each matrix consisted of 12 three-digit numbers (e.g., 4.69; see Mazar et al., 2008). Participants were given 5 min to find the two numbers in each matrix that equaled 10.00 when summed. The time limit was insufficient to allow solving of all 20 matrices (Mazar et al., 2008; Gino et al., 2009). Participants were instructed that they would earn NT $20 (approximately US $0.66) for each correct solution.

A stopwatch notified the participants when 5 min had passed. Participants folded their worksheets and placed them in a recycling box positioned in the corner of the room. A specific three-digit number in the example matrix was matched to each participant to allow us to examine their actual performance. Participants then counted how many matrices they had solved and paid themselves for their performance. An envelope containing 20 NT $20 coins was placed on the desk before the participants arrived; they were instructed to take one coin for every matrix that they had solved correctly. The self-pay arrangement was designed to provide an opportunity for cheating. The number of coins taken by each participant represented their self-scored performance. The overstated performance (i.e., the indicator of cheating) was calculated as the difference between self-scored performance and actual performance.

### Results

There were no group differences in ratings of contextual detail [*F*_(2,87)_ = 2.095, *p* = 0.129] and vividness [*F*_(2,87)_ = 1.321, *p* = 0.272]. **Table 2** shows that the discounting rate differs among the three study conditions [*F*_(2,87)_ = 4.868, *p* = 0.01, $\eta_{p}^{2}$ = 0.101]. Planned contrasts indicated that participants engaging in EFT pertaining to the ideal self were less likely to discount the future than were those in the control ET (*t* = -2.522, *p* = 0.013, *d* = 0.65) and ET pertaining to the present self (*t* = -2.852, *p* = 0.005, *d* = 0.74) groups. The discounting rate did not differ between the latter two groups (*t* = -0.329, *p* = 0.743). As shown in **Table 2**, there were no significant differences in actual performance on the matrix task across the three conditions [*F*_(2,87)_ = 1.698, *p* = 0.189]. However, self-scored performance varied as a function of study condition [*F*_(2,87)_ = 8.279, *p* = 0.001, $\eta_{p}^{2}$ = 0.16]. Participants engaging in EFT pertaining to the ideal self claimed to have correctly solved less matrices than did those under the control ET (*t* = -3.47, *p* = 0.001, *d* = 0.90) and the ET pertaining to the present self (*t* = -3.575, *p* < 0.001, *d* = 0.92) conditions. Self-scored performance did not differ between our two control conditions (*t* = -0.105, *p* > 0.05).

**Table 2:** Descriptive statistics by condition in Experiment 2.

Measure	EFT ideal-self		Control ET		ET present-self	
			
	*M*	*SD*	*M*	*SD*	*M*	*SD*
Discounting rate	0.44	0.24	0.59	0.22	0.61	0.23
Actual performance	7.23	2.00	8.07	1.74	7.97	1.99
Self-scored performance	8.17	2.32	10.37	2.74	10.43	2.29
Cheating	0.93	1.96	2.30	2.63	2.47	2.65


*Each condition involved 30 participants. Smaller values of the discounting rate represents lower discounting. The measure of cheating was defined as the difference between the number of correctly solved matrices claimed in the self-scored performance and the actual performance (i.e., overstated performance) in the matrix task. EFT,episodic future thinking; ET, episodic thinking.*

Moreover, we found that cheating was associated with the experimental condition [*F*_(2,87)_ = 3.583, *p* = 0.032, $\eta_{p}^{2}$ = 0.076]. **Table 2** shows that the mean difference between self-scored and actual performance (i.e., the overstated performance) was significantly smaller among participants under the EFT ideal-self condition than among those under the control ET (*t* = -2.174, *p* = 0.032, *d* = 0.56) and ET present-self (*t* = -2.439, *p* = 0.017, *d* = 0.63) conditions. The overstated performance did not differ between the two control conditions (*t* = -0.245, *p* > 0.05). A similar pattern was observed with regard to the percentage of participants who overstated their performance with the aim of obtaining undeserved reward [χ^2^_(2, N = 90)_ = 7.216, *p* = 0.021, Nagelkerke *R*^2^ = 0.105]. Two dummy variables (EFT ideal-self vs. control ET; ET present-self vs. control ET) were created for our three study conditions, treating the control ET condition as the reference group. A logistic regression showed that participants under the EFT ideal-self condition were less likely to cheat in the matrix task (20.0%, 6 out of 30) than were those under the control ET condition (46.7%, 14 out of 30; *B* = -1.25, *SE* = 0.59, *p* = 0.032, Wald = 4.585, Odds Ratio = 0.29, 95% CI: 0.09–0.90); however, the likelihood of cheating did not differ between the ET present-self condition (50.0%, 15 out of 30) and the control ET condition (*B* = 0.13, *SE* = 0.52, *p* = 0.796).

Additionally, we examined whether delay discounting mediated the link between the thinking manipulation and cheating. As the control EFT and ET present-self groups did not differ in both the discounting rate and level of cheating, these two groups were combined as the reference group for the dummy variable (1 = EFT ideal-self, 0 = control ET and ET present-self). The relationship between engagement in EFT pertaining to the ideal self and the likelihood of cheating (*B* = -1.32, *SE* = 0.52, *Z* = -2.516, Wald = 6.331, *p* = 0.012) was no longer significant (*B* = -6.97, *SE* = 10.61, *Z* = -0.657, Wald = 0.431, *p* = 0.511) after controlling for the discounting rate. A bootstrap analysis showed that the indirect effect was significant (*B* = -9.72, *SE* = 3.32, 95% bias-corrected CI: -15.20 to -2.29; bootstrap resamples = 5000). Thus, the mediation analysis results suggest that lower discounting, induced by engaging in EFT with a focus on the ideal self, leads to refraining from cheating.

### Discussion Experiment 2

In summary, the results of Experiment 2 were consistent with those of Experiment 1, and provide support for the hypothesized connection between engagement in EFT pertaining to the ideal self and a reduced tendency toward delinquency. The mediation analysis suggested that EFT pertaining to the ideal self reduces the likelihood of cheating by shifting the time perspective of intertemporal decision-making, that is, a lower tendency toward discounting. However, the size of the indirect effect in the mediation model was moderate. It should be noted that other intervening variables (e.g., self-control, Baumeister et al., 2007) may also have affected the observed association between EFT about the ideal self and the tendency toward cheating.

## General Discussion

We conducted two experiments to demonstrate that engagement in EFT pertaining to the ideal self leads to lower discounting rates (Experiments 1 and 2), in turn associated with a decreased tendency toward delinquent choices in imaginary scenarios (Experiment 1) and a reduced likelihood of cheating in a matrix task (Experiment 2). The finding regarding the effect of using EFT to envisage future life events (experienced by the ideal self) on delay discounting is congruent with recent research on methods for overcoming the discounting tendency (e.g., abstract processing, Chiou et al., 2013; enhanced connections to the future self, Hershfield, 2011; and vividly imagining the future self, van Gelder et al., 2013; Kuo et al., 2016). Our results indicate that engaging in EFT to pre-experience life events as the ideal self effectively lowers the discounting rate by promoting emphasis on the distant costs of delinquent acts; thus, the temptation to engage in delinquent behavior is more likely to be resisted. In other words, whether engaging in EFT pertaining to the ideal self leads to reduced delinquency may hinge on the inclination to adopt a future-oriented focus, that is, a lower tendency toward discounting. These results have an important practical implication, namely, that EFT interventions focusing on the ideal self could reduce the propensity toward delinquency.

The current research complements the existing literature by showing that engaging in EFT pertaining to the ideal self is associated with a reduced tendency toward delinquency. The mediation analysis suggested that our EFT manipulation could foster a more “future-oriented self,” leading to greater consideration of the long-term costs of delinquent behavior. These findings are in accordance with the active-self account of priming effects (Wheeler et al., 2007), which proposes that an activated mental representation (e.g., placing more emphasis on the distant costs of delinquency in the current context) mediates prime-to-behavior effects (Ferguson and Bargh, 2004; Loersch and Payne, 2011). For example, experiencing brightness may heighten the salience of moral considerations pertaining to the self, thereby leading to ethical behavior (Chiou and Cheng, 2013). Furthermore, wearing counterfeit clothing could lead individuals to experience a “counterfeit self,” such that their likelihood of behaving dishonestly will be increased (Gino et al., 2010). Similarly, the use of cheaper, generic products causes people to experience a devalued self that leads to unfavorable self-evaluations (Chiou and Chao, 2011). The connection that we observed between delay discounting and the tendency toward delinquency accords well with Gottfredson and Hirschi’s (1990) general theory of crime, indicating that low self-control, as manifested in greater discounting, is an important predictor of delinquency.

The present findings contribute to the existing literature in several important ways. First, we provide convergent evidence that participants will show lower rates of discounting after engaging in EFT pertaining to their ideal selves, suggesting an increased likelihood that they will consider the long-term consequences of delinquent acts. Second, the observed link between an ideal self-related EFT intervention and a decreased tendency toward delinquent choices and cheating indicates that mental simulation of future life events experienced by the ideal self may help individuals resist impulses to engage in morally questionable behavior. Finally, we found that delay discounting mediated the connection between engagement in EFT pertaining to the ideal self and the tendency toward virtual delinquent choices. The findings suggest that taking account of the tendency toward discounting may be instructive in interventions for delinquency reduction or prevention.

With respect to limitations and future directions, our two experiments involved college students, and the findings were obtained in a laboratory setting and represented immediate effects only. Thus, caution should be exercised when inferring generalizability. Long-term investigations of the temporal duration and persistence of the effects of the intervention are needed to confirm its practical utility. Discounting rates were assessed in the monetary-choice task; alternative methods of measuring discounting tendency, such as the area under the curve (Myerson et al., 2001) and the non-monetary discounting task (e.g., Hardisty and Weber, 2009; Joshi and Fast, 2013), should be employed for convergent validation. Although a non-intervention control group (included in Experiment 1) provided a delay discounting baseline, a pre- and post-test design would allow assessment of the effectiveness of EFT interventions in reducing the tendency toward discounting. Additionally, we acknowledge that the lack of an EFT-alone condition in Experiment 2 is a limitation. Indeed, questions about whether engagement in EFT about the future ideal self would be more beneficial for reducing the tendency toward cheating than would engagement in EFT about the future more generally remain unanswered. It is possible that engagement in EFT *per se* might reduce cheating.

Our findings also suggest several interesting avenues for future research. Given that control over delinquent acts requires the ability to delay gratification across diverse contexts, as well as over time, can EFT focusing on the ideal self also be effective in regulating other impulse control-related behaviors, such as compulsive Internet use, overeating (i.e., weight control), drug and alcohol use, gambling, and problematic online or offline video gaming? It would also be instructive for future research to examine specifically the relationship between EFT involving a particular aspect of the ideal self (e.g., a weight-reduced self) and its corresponding impulse-control behavior (e.g., dietary practices or exercising). Moreover, we suspect that the delinquency-reducing effect of EFT pertaining to the ideal self is not invariant. Temporal distance has been shown to affect individuals’ ability to imagine future events (Trope and Liberman, 2003, 2010; D’Argembeau and Van der Linden, 2004). Given that the thinking exercise in the present research focused on a particular time period, future research could examine whether temporal distance moderates the effect of envisaging life events as experienced by the ideal self on delinquency. A connection to the future self has been show to play a crucial role in individuals’ conceptualization of that future self (Hershfield, 2011). Whether individuals with high future self-continuity are more receptive to our EFT intervention is especially worthy of investigation.

## Conclusion

The current research demonstrated that engagement in EFT about the ideal self could undermine the tendency toward virtual delinquent choices and the likelihood of cheating. Given that successfully refraining from delinquent acts requires individuals to resist immediate benefits and focus on distant costs, an EFT intervention involving the ideal self may promote delinquency prevention by influencing the tendency to delay gratification. Educators could incorporate EFT interventions into moral- and character-based exercises to help students achieve a “better self.” Although people often yield to the temptation of instant rewards, and fail to think through the future consequences of morally questionable behavior, future-oriented individuals are less likely to discount temporally remote consequences (Nagin and Pogarsky, 2003; van Gelder et al., 2013). This study indicates that engagement in EFT with a focus on the ideal self can help people to promote delayed gratification. To fight the impulse toward the immediate benefits of delinquent acts, it may be of benefit to engage frequently in prospective imagery, that is, to envisage future life events as experienced by the ideal self.

## Ethics Statement

This research protocol was approved by the research ethical committee of the Kaohsiung Medical University. The participants signed an informed consent form stating the duration of the study and explaining they could withdraw from the study, and that the data would be anonymous.

## Author Contributions

W-HW, WC, and W-BC designed the study described in the manuscript and supervised the data collection. W-HW and W-BC participated in the data collection. WC and W-BC performed the analyses. W-BC wrote the first draft of the paper. The first draft of the paper was revised by W-HW and WC.

## Conflict of Interest Statement

The authors declare that the research was conducted in the absence of any commercial or financial relationships that could be construed as a potential conflict of interest.
